# Effect of estradiol and predator cues on behavior and brain responses of captive female house sparrows (*Passer domesticus*)

**DOI:** 10.3389/fphys.2023.1172865

**Published:** 2023-06-23

**Authors:** Melanie G. Kimball, Courtney T. Harding, Kaitlin E. Couvillion, Keegan R. Stansberry, Tosha R. Kelly, Christine R. Lattin

**Affiliations:** Department of Biological Sciences, Louisiana State University, Baton Rouge, LA, United States

**Keywords:** predator playback, anti-predator behavior, immediate early gene, ZENK, *Passer domesticus*

## Abstract

The presence of predators can cause major changes in animal behavior, but how this interacts with hormonal state and brain activity is poorly understood. We gave female house sparrows (*Passer domesticus*) in post-molt condition an estradiol (n = 17) or empty implant (n = 16) for 1 week. Four weeks after implant removal, a time when female sparrows show large differences in neuronal activity to conspecific vs. heterospecific song, we exposed birds to either 30 min of conspecific song or predator calls, and video recorded their behavior. Females were then euthanized, and we examined neuronal activity using the expression of the immediate early gene (IEG) ZENK to identify how the acoustic stimuli affected neuronal activation. We predicted that if female sparrows with estradiol implants reduce neuronal activity in response to predator calls as they do to neutral tones and non-predatory heterospecifics, they would show less fear behavior and a decreased ZENK response in brain regions involved in auditory (e.g., caudomedial mesopallium) and threat perception functions (e.g., medial ventral arcopallium) compared to controls. Conversely, we predicted that if females maintain auditory and/or brain sensitivity towards predator calls, then female sparrows exposed to estradiol would not show any differences in ZENK response regardless of playback type. We found that female sparrows were less active during predator playbacks independent of hormone treatment and spent more time feeding during conspecific playback if they had previously been exposed to estradiol. We observed no effect of hormone or sound treatment on ZENK response in any region of interest. Our results suggest that female songbirds maintain vigilance towards predators even when in breeding condition.

## 1 Introduction

Predation risk often induces measurable changes in animal behavior, and animals often respond to predator calls differently than other types of sounds. For example, in response to predator calls, black-capped chickadees (*Poecile atricapillus*) attempted to recruit nearby conspecifics to mob predators, frequently moved from location to location, and did not ruffle their feathers (Congdon et al., 2016). Researchers interpreted these responses as preparation to attack, to elude the predator, and to reduce the risk of being seen in high-threat conditions, respectively. Increased freezing and vigilance in response to predator cues are commonly observed across taxa (Beani and Dessí-Fulgheri, 1998; Mesquita and Young, 2007; Rahlfs and Fichtel, 2010). In the chickadee brain, exposure to predator vocalizations induced neural activation in areas involved in fear and emotion (AMV and NCL) (Hobbs, 2015; Zanette et al., 2019). These differences in brain activity may be correlated with behavioral response to predators, but this has rarely been investigated outside of mammalian systems (but see Cross et al., 2013). Further, we do not fully understand how an animal’s underlying hormonal state affects anti-predator behaviors and neuronal response to predators.

Previous research has shown that hormonal state can affect both the production of and the neural response to breeding signals like mating calls. Efficient auditory communication requires the rapid recognition of salient signals and the ability to filter other sounds. The preference for conspecific vs. heterospecific song has been well characterized in songbirds, and conspecific-selective neuronal activity has been found in auditory nuclei like the caudomedial mesopallium (CMM) and the caudomedial nidopallium (NCM) (Mello et al., 1992; Louder et al., 2019). Sex steroids may influence auditory processing by affecting both auditory and social behavior brain regions (e.g., the medial ventral arcopallium) (Maney and Pinaud, 2011), by altering the auditory brainstem response (Caras et al., 2010), or even by acting on the ear directly (Noirot et al., 2009). Estradiol increases the range of frequency sensitivity of the hearing organs of some animals, likely increasing female sensitivity to male vocalizations (Sisneros and Bass, 2003; Ronald et al., 2018), Estradiol can directly modulate the selectivity of neural substrates due to the abundance of estrogen production throughout the avian brain (Vahaba and Remage-Healey, 2018) or via circulating estrogen produced in the ovary (Maney et al., 2007), and there is a high density of estrogen receptors in many relevant brain nuclei (Bernard et al., 1999) as well as in the auditory brainstem and ear (Henry and Lucas, 2009). For example, female songbirds in breeding condition increase neuronal activity in response to conspecific song compared to frequency-matched tones in several brain regions of the social behavior network (Maney et al., 2008; Maney and Pinaud, 2011; O’Connell and Hofmann, 2011). House sparrows with experimentally-increased estradiol showed decreased neuronal activity to heterospecific compared to conspecific song in brain regions involved in auditory perception (Lattin et al., 2017b). Additionally, when plasma estradiol reached breeding levels, activity in the auditory forebrain of female white-throated sparrows (*Zonotrichia albicollis*) selected for conspecific song, suggesting that processing of auditory conspecific signals is seasonally regulated by sex steroids (Sanford et al., 2010). However, past studies have focused solely on comparisons between conspecific song and neutral acoustic stimuli (i.e., tones or the song of other songbirds). What is unknown is whether female songbirds in breeding condition also “tune out” the calls of predator species like hawks and owls that might present a danger to them; i.e., do females maintain sensitivity to predatory stimuli while in breeding condition? It is undeniably important to maintain vigilance towards predation no matter the time of year. However, several studies have reported increased depredation of female songbirds during the breeding season (Slagsvold and Dale, 1996; Götmark et al., 1997; Post and Götmark, 2006), which could, in part, be explained by a decrease in auditory and neuronal sensitivity to predator calls.

In this experiment, we were interested in testing two competing hypotheses: 1) Hormonal state affects neuronal response to predator cues; 2) Hormonal state does not affect neuronal response to predator cues (hereafter referred to as the alternative and null hypotheses, respectively). To test these hypotheses, we examined both behavior and protein expression of the immediate early gene (IEG) ZENK as a measure of neuronal activity in captive female house sparrows (*Passer domesticus*) exposed to predator calls or male conspecific song. ZENK is often used to assess neuronal responses to acoustic stimuli in songbirds (Maney et al., 2008; Lynch et al., 2012; Rivera et al., 2019), therefore we interpret ZENK immunoreactivity as neuronal activity. The caudomedial nidopallium (NCM, involved in auditory perception), caudomedial mesopallium (CMM, involved in auditory memory), medial ventral arcopallium (AMV, involved in threat perception), caudal hippocampus (cHP, involved in integrating sensory and emotional responses), apical hyperpallium (HA, involved in behavioral flexibility), and caudolateral nidopallium (NCL, involved in decision making) all increase neuronal activity in response to aversive or threatening conditions (Rose and Colombo, 2005; Cross et al., 2013; Dai et al., 2018; Brito et al., 2019; Zanette et al., 2019; Kimball et al., 2022). These regions are therefore ideal candidates to respond to predator calls. Because estradiol influences auditory processing, we simulated breeding condition in half of the females with estradiol implants and the other half received empty implants as a control.

To understand how reproductive status modulates responsiveness to predator cues relative to conspecific cues, we first analyzed behavior during acoustic exposures. Based on previous literature, we expected to see higher levels of freezing in female sparrows exposed to predator calls compared to females exposed to male conspecific song. However, if estradiol-treated females showed reduced responsiveness to predator calls, we predicted less freezing behavior in this group compared to predator-exposed females receiving empty implants. We then analyzed neural responses. If females in breeding condition maintain sensitivity to predator calls, we predicted female sparrows exposed to estradiol would have no difference in ZENK response in any region of interest regardless of playback type (i.e., predator or conspecific calls). If female sparrows exposed to estradiol decrease responsiveness to predator calls, as previously observed with heterospecific calls and neutral tones, we predicted they would have lower ZENK response in brain regions involved in auditory perception (e.g., NCM and CMM) compared to estradiol-treated females hearing male sparrow song. We also expected to see less ZENK response in regions involved in aversive responses and threat perception (AMV, NCL, cHP, and apical hyperpallium) in estradiol-treated sparrows hearing predator calls compared to females with empty implants. We predicted no difference in ZENK response between conspecific and predator playback in sparrows receiving empty implants because the NCM and CMM showed increased selectivity towards conspecific signaling only when plasma estradiol reached breeding levels (Maney et al., 2006; Sanford et al., 2010).

## 2 Materials and methods

### 2.1 Study subjects

Adult female house sparrows (n = 33) were captured using mist nets at bird feeders in East Baton Rouge Parish between June and August 2020. Sparrows were doubly housed in cages in a vivarium at Louisiana State University with unlimited access to mixed seeds, grit, a vitamin-rich food supplement (Purina Lab Diet), and water. Sparrows also had access to a variety of perches and a dish of sand for dustbathing. Sparrows were maintained at natural day length (13L:11D) for a minimum of 4 weeks to acclimate to the captive environment before implant surgeries began. Although females were kept on long days, all females molted in captivity and females in the control group had small ovaries (data not shown), suggesting females were photosensitive but not photostimulated before receiving implants. Animals were collected under a Louisiana State Scientific Collecting Permit and all experimental procedures approved by the Louisiana State University Institutional Animal Care and Use Committee under protocol 96-2018. We used approved methods for bird capture, transport, husbandry, and surgery as specified in the Ornithological Council’s Guidelines to the Use of Wild Birds in Research (Fair et al., 2010), and approved methods of euthanasia for laboratory animals as specified in the 2020 American Veterinary Medical Association Guidelines for the Euthanasia of Animals.

### 2.2 Implant surgeries

All sparrows received subcutaneous implants in the skin of the back (n = 17 estradiol, n = 16 control). Estradiol implants consisted of silastic medical-grade tubing packed with crystalline 17-beta-estradiol (Sigma-Aldrich, St. Louis, MO, United States), sealed at both ends with a silicone adhesive. Control implants were empty. The size of implants (15 mm long, 2 mm outer diameter) was the same as used in a previous experiment (Lattin et al., 2017b), which were shown to significantly increase ovary size (437.6 ± 102.5 mg) compared to females with empty implants (13.4 ± 12.3 mg). Estradiol implants also significantly increased levels of circulating estradiol concentrations to (2.5 ± 0.7 ng/ml) compared to females with empty implants (0.16 ± 0.05 ng/ml) and 4 weeks after implant removal (1.1 ± 0.8 ng/ml) (Lattin et al., 2017b).

For implant surgeries, sparrows were anesthetized with inhaled isoflurane (4% induction, 3.5%–2% maintenance), and maintained at a surgical plane of anesthesia. Depth of anesthesia was assessed using toe pinch, breathing rate, and palpebral reflex, and we used a heating pad under a sterile surgical pad to maintain body temperature. Birds were given subcutaneous ketoprofen (5 mg/kg) as a pre-emptive analgesic, a small incision was made in cleaned and disinfected skin between the shoulder blades, an implant was inserted, and the incision site closed with Vetbond (3M, Maplewood, MN, United States). The following day, all birds were monitored to ensure proper healing and check implant placement and were given a second dose of ketoprofen to minimize discomfort. Implants remained in place for 1 week and were then removed using a similar procedure to implant insertion: isoflurane anesthesia (4% induction, 3.5%–2% maintenance), ketoprofen, an incision in cleaned and disinfected skin, removal of the implant using sterile forceps, and the incision site closed with Vetbond. Sparrows were checked again the day after implant removal to ensure proper healing and given a final dose of ketoprofen. Four weeks later, we conducted playback experiments. We used this time course because in a previous house sparrow study, greater effects of estradiol treatment on brain responses to conspecific vs. heterospecific playback were observed during this later time point than during the first week implants were in place (Lattin et al., 2017b), suggesting that the full effects of estradiol on the brain and auditory perception can take several weeks to fully develop.

### 2.3 Playback trials

The evening before playback trials, females were individually housed in smaller test cages with access to multiple perches, food, grit, and water. For playback trials, females were rapidly transported one at a time to an acoustically isolated testing room and exposed in individual trials to either a unique 30 min playlist of several different male house sparrows singing with a few calls (n = 8 control females, n = 8 estradiol-treated females) or a unique mix of calls from local predators: Barn owls (*Tyto alba*), Eastern screech owls (*Megascops asio*), Great horned owls (*Bubo virginianus*), Broad-winged hawks (*Buteo platypterus*), Cooper’s hawks (*Accipiter cooperii*), Red-shouldered hawks (*Buteo lineatus*), Red-tailed hawks (*Buteo jamaicensis*), American kestrels (*Falco sparverius*), Loggerhead shrikes (*Lanius ludovicianus*), and Mississippi kites (*Ictinia mississippiensis*) (n = 8 control females, n = 9 estradiol-treated females). Therefore, treatment groups were as follows: n = 8 for Empty + Predator, n = 8 for Empty + Sparrow, n = 9 for Estradiol + Predator, and n = 8 for Estradiol + Sparrow. A female sparrow heard, on average, 31.7 ± 1.5 (range: 30–35) different male house sparrow sound files during conspecific playbacks or 58.5 ± 2.9 (range: 53–63) different predator sound files during predator playbacks. All 11 predator species were represented at least once in each predator playback playlist, and each had on average five different songs or calls per sound file. Sound files were obtained from the Macaulay Library (Cornell Lab of Ornithology, Ithaca, NY, United States) and the Borror Laboratory of Bioacoustics (The Ohio State University, Columbus, OH, United States). Each bird heard a unique playlist of different sound files played in a randomized order. No other sparrows were present in the testing room during trials. Loudness was standardized to 60 dBA from bird to speaker using a sound level pressure meter and we video recorded female behavior during the playback trials using a Logitech C615 portable webcam. A researcher began recording videos after transporting the individual cage, and then started the playback and immediately exited the room.

Previous work in songbirds has shown that IEG proteins peak ∼90 min after stimulus exposure (Goodson et al., 2005). Therefore, after the 30 min playback behavior trials, females were transported to a dark quiet room for 60 min before being deeply anesthetized with ketamine (80 mg/kg) and xylazine (20 mg/kg), doses shown to be appropriate for house sparrows (Muresan et al., 2008). Once animals were in a surgical plane of anesthesia, they were transcardially perfused with ice-cold heparinized saline and 0.1 M phosphate buffer containing 4% paraformaldehyde sequentially. Euthanasia was confirmed using rapid decapitation and brains extracted.

### 2.4 Behavioral analysis

Thirty-minute video recordings were analyzed using BORIS v 7.10.2 (Friard and Gamba, 2016). One individual’s video was lost due to a recording malfunction, therefore final sample sizes for behavior were as follows: n = 8 for Empty + Predator, n = 8 for Empty + Sparrow, n = 9 for Estradiol + Predator, and n = 7 for Estradiol + Sparrow. An ethogram was created to associate keys with point-type behaviors, which were discrete behaviors with no duration (movement, beak wiping, feather ruffling, and calling), and state-type, which were behaviors with duration (preening and feeding). We were interested in any behaviors that might have been altered by threatening stimuli. Movement was classified as a hop, flight, jump, or any time both feet came off the ground. Behaviors like foot adjustments, shuffling, stretching, and head bobbing were not considered movements. Beak wiping was counted when an individual wiped its beak on an object in the cage, usually a perch. One bout of beak wiping was considered when at least 2 s occurred between subsequent wipes (Lattin et al., 2017a). Calls were classified as one bout of vocalizations (e.g., chirp or rattle). Feather ruffling was classified as an event when an individual would puff up its feathers and ruffle quickly. Preening was classified as any time an individual would pull on their feathers with their beak or spread oil from their preen glands. Finally, feeding was classified when the bird perched on the food dish in the cage and fed. For each individual, the total number of occurrences were recorded for each behavior and total duration was recorded for state-type behaviors and means and standard deviations were calculated for each treatment group. Videos were watched without sound to classify all behaviors except for calls, so the observer was blind to playback type (predator vs. male sparrow), and these videos were watched by the same observer to ensure consistency (intra-observer coefficient of variation from watching 4 videos twice: movement = 0.5%, feeding duration = 1.1%). A separate observer watched all videos with the sound on to be able to accurately quantify sparrow calling behavior. Both observers were blind to bird treatment (estradiol vs. empty implant). Beak wiping, feather ruffling, calls, and preening were infrequently observed (each occurred in 25% or fewer of videos) and were not included in the final behavior analysis.

### 2.5 Immunohistochemistry and ZENK quantification

Brains were post-fixed in 4% paraformaldehyde phosphate buffer for 24 h at 4°C, then soaked in 0.1 M phosphate buffer containing 30% sucrose for cryoprotection. After sinking (∼2 days), brains were flash-frozen in powdered dry ice and stored at −80°C until sectioning. Brains were cut at −20°C in the coronal plane in 40 µm sections using a ThermoFisher NX50 cryostat. Starting at striatum, triplicate sections were collected in wells containing cryoprotectant (0.2 M phosphate buffer, 15 M PVP, 1.5 M sucrose, and 0.5 M ethylene glycol in distilled water) and stored at −20°C until the day of immunohistochemistry.

Brain regions were identified based on visible landmarks. Apical hyperpallium (HA) sections were taken when Area X was still visible and approximately ∼120 µm before the first appearance of the lateral septum. We used caudal dorsomedial hippocampal sections where the cerebellum first became visible and the mesopallium began to disappear. We targeted medial ventral arcopallium (AMV) based on the visibility of the cerebellum and arcopallium. Sections used for caudolateral nidopallium (NCL) were 40 µm after AMV sections, in a pallial area where we have confirmed the presence of dense basket fiber staining for tyrosine hydroxylase in house sparrows, consistent with NCL in other songbird species (von Eugen et al., 2020). Caudomedial mesopallium (CMM) was taken ∼40–80 µm before and caudomedial nidopallium (NCM) was taken ∼40–80 µm after the appearance of field L2. For each region we ran immunohistochemistry for all 33 animals in the same assay on the same day.

Sections were stained for ZENK as done previously (Kimball et al., 2022). Briefly, sections were washed 3 times in Tris-buffered saline (TBS, pH 7.6), incubated in 0.5% hydrogen peroxide for 30 min, washed again 3 times in TBS, and blocked with 10% normal horse serum (Vector Laboratories, Burlingame, CA, United States of America) in 0.3% Triton in TBS (TBS-T) for 1 h. After washing 3 more times in TBS, sections were moved out of mesh well inserts and incubated with a monoclonal mouse anti-ZENK antibody (1:500 in TBS-T and 1% normal horse serum; antibody 7B7-A3) donated by Dr. David Keays, Research Institute of Molecular Pathology in Vienna, Austria and raised against an N-terminal fragment 260 amino acids in length (1–260) of rock pigeon ZENK (Nordmann et al., 2020). Sections were incubated for ∼20 h at 4°C. After washing 3 times in TBS, sections were then incubated at room temperature for 1 h in biotinylated horse anti-mouse IgG (Vector Laboratories) diluted 1:500 in TBS-T, followed by three more washes in TBS-T. Sections were incubated in avidin–biotin horseradish-peroxidase complex (Vectastain ABC, Elite kit, Vector) at a concentration of 1:100 for 1 h and washed twice in TBS. Sections were visualized with DAB (Sigma Fast-DAB), mounted onto slides, dehydrated in ethanol, cleared in HemoDe (Scientific Safety Solutions, Keller, TX, United States), and cover-slipped using Permount (Electron Microscopy Sciences, Hatfield, PA, United States).

**FIGURE 1:**
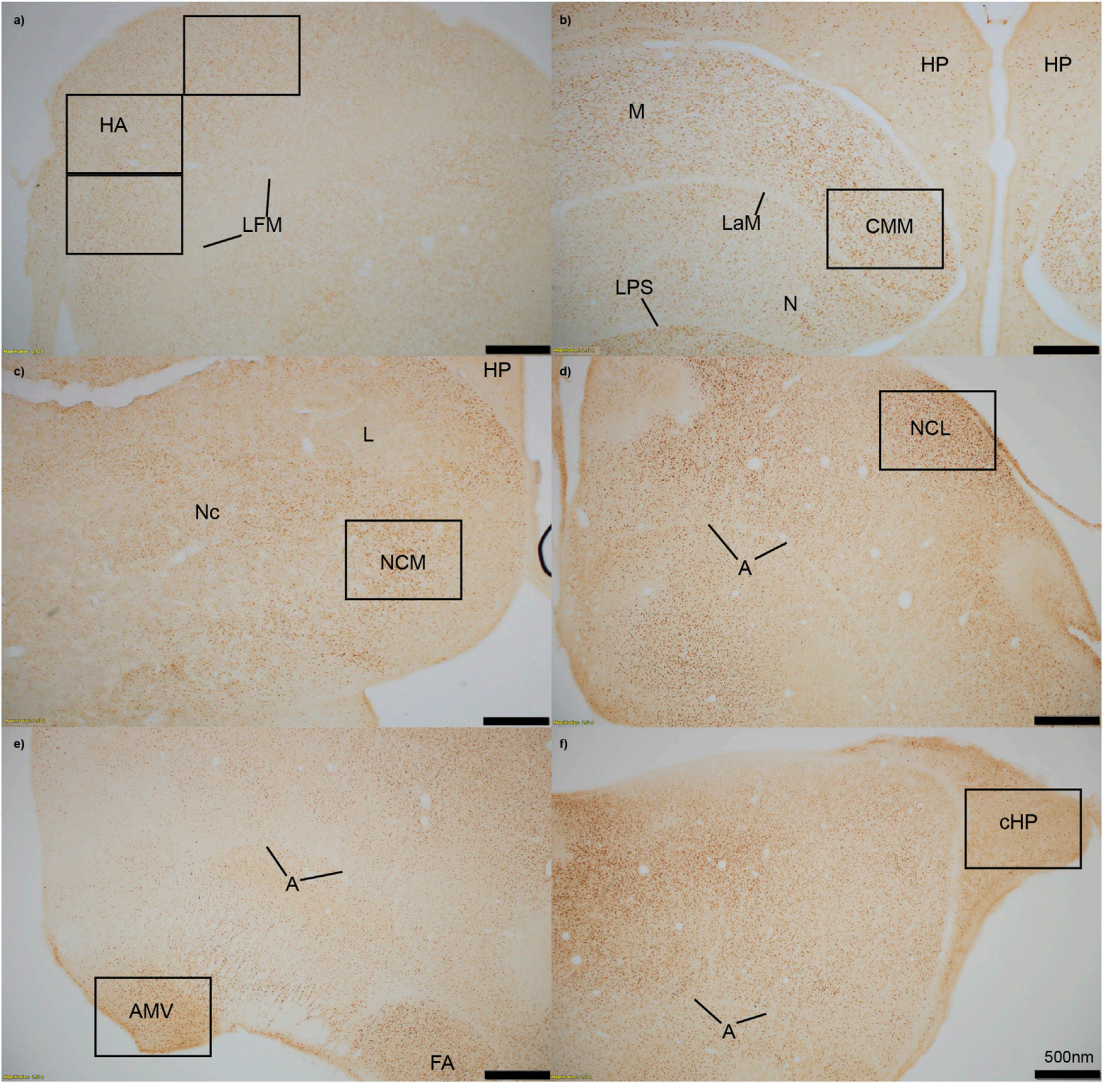
Immediate early gene ZENK staining in six regions of interest at 4x magnification. **(A)** HA = apical hyperpallium, **(B)** CMM = caudomedial mesopallium, **(C)** NCM = caudomedial nidopallium, **(D)** NCL = caudolateral nidopallium, **(E)** AMV = medial ventral arcopallium, **(F)** cHP = caudal hippocampus. Quantification of active neurons in each region occurred in black boxes, which approximately represent 20x magnification. Stereotaxic coordinates are not labelled because there is currently no atlas for the house sparrow brain. A = arcopallium, FA = tractus fronto-arcopallialis, HP = hippocampus, L = field L, LaM = lamina mesopallialis, LFM = lamina frontalis suprema, LPS = lamina pallio-subpallialis, M = mesopallium, N = nidopallium, Nc = nidopallium caudale.

We measured immunopositive cell density for ZENK in all six regions of interest. Images of each region were captured using an Olympus TH4-100 microscope with a ×20 objective lens using consistent lighting for each photo. We quantified four sections per individual, with each section including right and left hemispheres unless the hemisphere was damaged during staining, measured the area that was quantified, calculated density (cells/mm^2^), and averaged the cell density for each individual in each region. One to two images per hemisphere were captured for smaller regions (cHP, CMM, NCM, NCL, and AMV), and three images were taken of the HA (Figure 1). Individuals were excluded from the following regions if more than two out of the four sections had unclear staining due to artifact, or if tissue was too torn to properly quantify cell density: cHP: n = 1 for Estradiol + Predator, n = 1 for Estradiol + Predator; HA: n = 1 for Empty + Predator, n = 2 for Empty + Sparrow, n = 1 for Estradiol + Predator; NCM: n = 1 for Empty + Sparrow, n = 1 for Estradiol + Predator, n = 2 for Estradiol + Predator; CMM: n = 1 for Empty + Predator, n = 1 for Estradiol + Predator, n = 2 for Estradiol + Sparrow.

We used ImageJ (Schneider et al., 2012) to measure immunopositive cell density in each image using a procedure adapted from Mischler et al. (2017). All images were cropped to include only the region of interest, and the area of each region was measured. We then converted each image to 8-bit grayscale, increased the contrast, and used the threshold tool to make immunopositive nuclei white against a black background. We then used the count function to quantify the number of immunopositive nuclei and calculated staining density. Image analysis was done by two observers blind to treatment (estradiol vs. empty and predator vs. sparrow).

### 2.6 Data analyses

We used JMP Pro 16.0 (SAS Institute) for all behavior and IEG analyses, and all individuals were included in each analysis (n = 33), unless data was missing (as described above). We first ran generalized linear models assessing the effect of hormone and sound treatments on behavior. Movement was Box-Cox transformed (Box and Cox, 1964), which fits variables to a normal distribution by creating a unique transformation calculation from the given raw data. The following equation was produced:

$$\frac{{\left(Movement+1\right)}^{0.255}-1}{0.00889}$$

Box-Cox transformation cannot be run on values of zero and below, therefore we added 1 to movement data prior to transformation. We used two separate generalized linear models to analyze sparrow behavior, with either movement or feed duration as the dependent variable and hormone treatment, sound treatment, and a hormone treatment * sound treatment interaction as fixed effects.

We next ran models assessing the effect of hormone and sound treatments on ZENK immunoreactivity in each region of interest (six models total), with ZENK density (cells/mm^2^) as the dependent variable and hormone treatment, sound treatment, and a hormone treatment * sound treatment interaction as fixed effects. In cases where there was a significant effect of treatment in behavior or IEG analyses, we compared treatment groups using ANOVA and Tukey’s HSD tests. For all models, Bartlett’s test indicated equal between-group variances. Normal quantile plots showed right-skewed data; therefore, all models were fit to an exponential distribution.

To investigate any possible associations between neuronal activity, movement, and feeding duration, we ran Spearman’s rank-order correlations. Spearman’s rank-order coefficients were calculated for average ZENK activity in the 6 brain regions of interest and the 2 behaviors, for a total of 12 correlations (e.g., ZENK activity in the AMV and movement). *p*-values in all analyses were corrected for multiple testing using the Holm-Bonferroni method (Holm, 1979).

## 3 Results

### 3.1 Behavior

**FIGURE 2:**
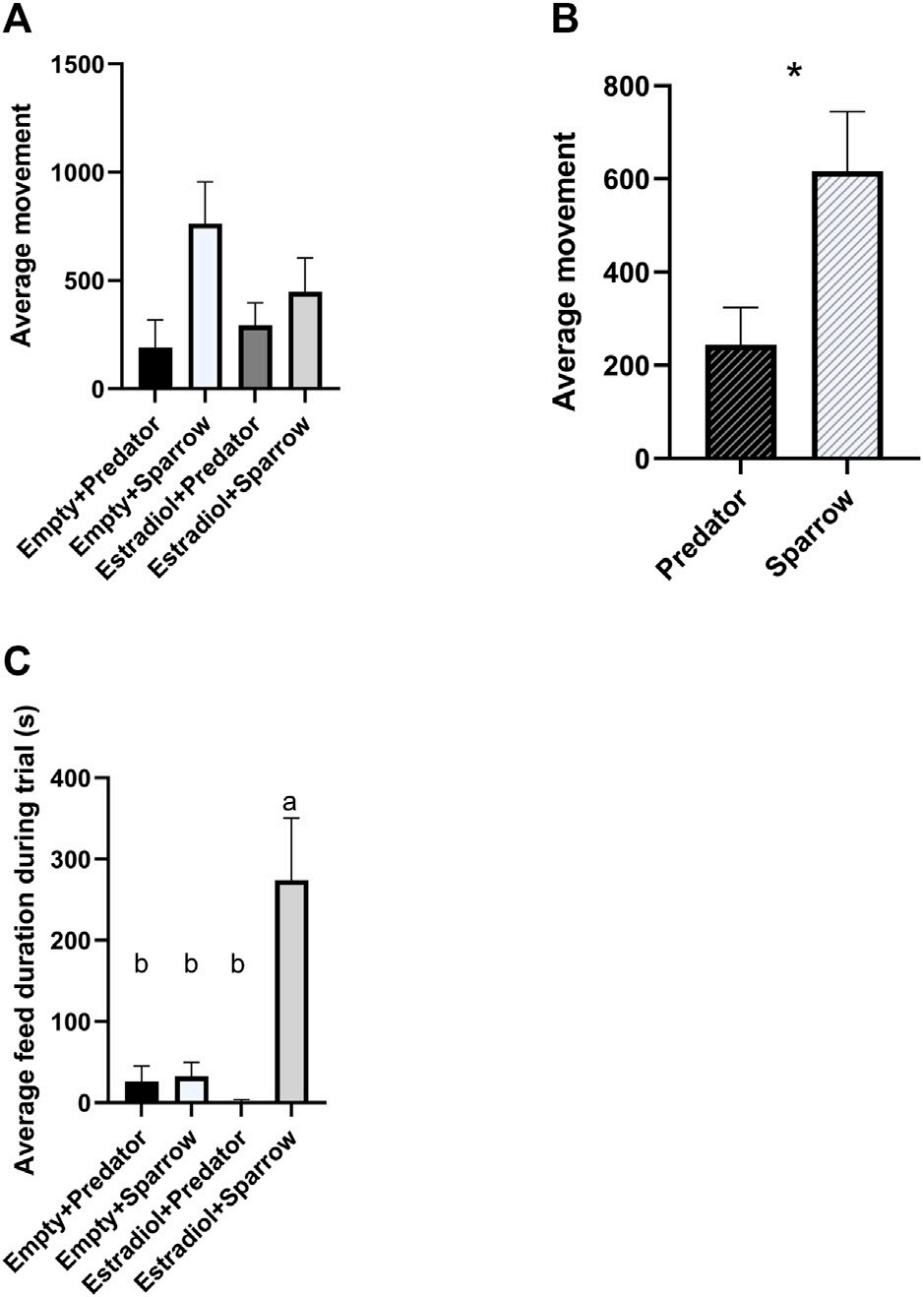
Female house sparrows moved less when exposed to predator calls **(A and B)** and fed more when treated with estradiol and exposed to conspecific song **(C)**. Behaviors were quantified for 30 min during playback. “Average movement” in panels a refers to the average number of hops and flights for females in that group. Sample sizes for panels a and c were n = 8 for Empty + Predator, n = 8 for Empty + Sparrow, n = 9 for Estradiol + Predator, and n = 7 for Estradiol + Sparrow. In panel b, sample sizes were n = 17 for Predator and n = 15 for Sparrow sound treatment groups. In panel c, different letters indicate *p* < 0.05. Error bars represent standard error. *p < 0.05.

**FIGURE 3:**
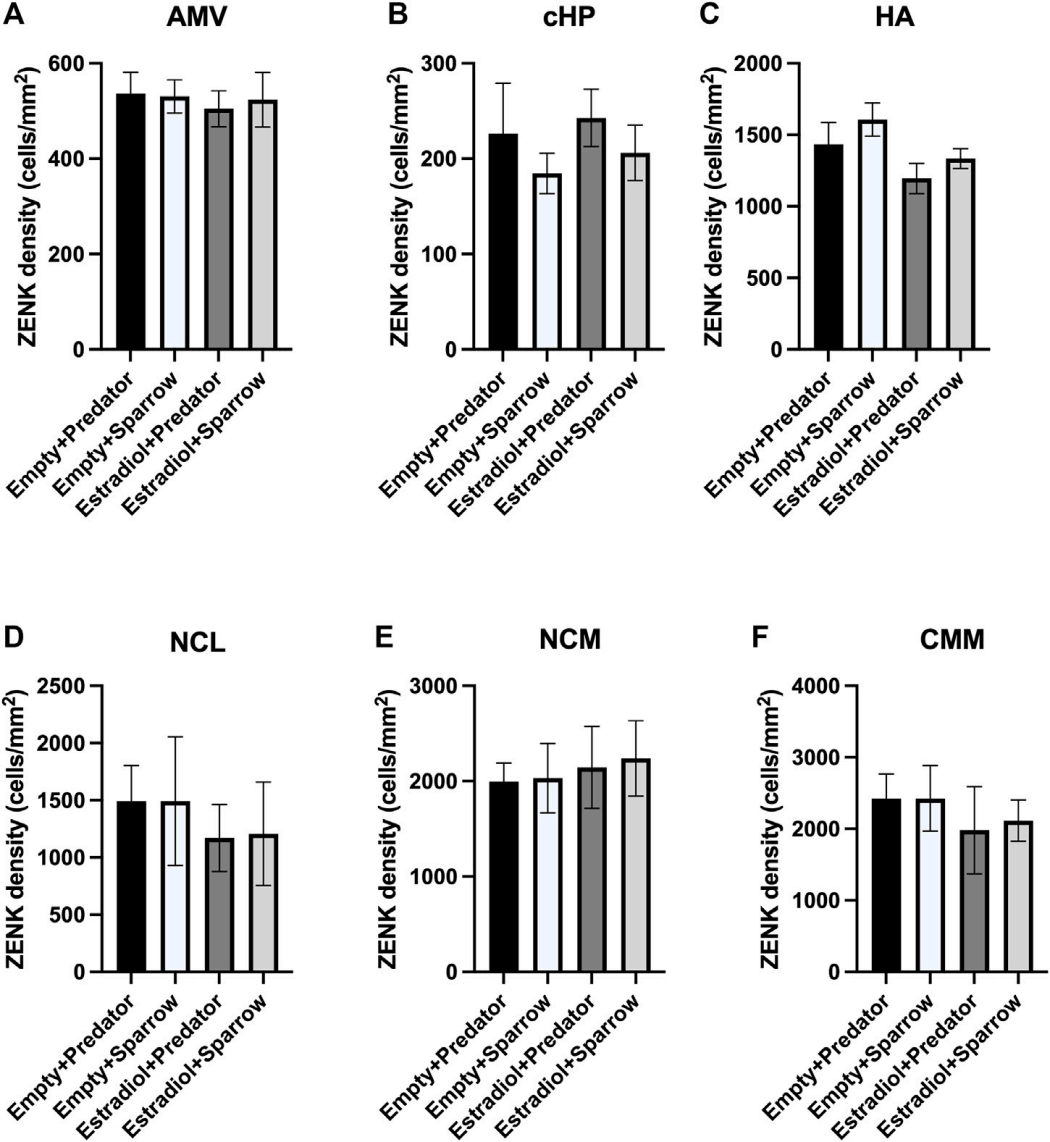
ZENK expression of female sparrows did not differ in response to predator or conspecific vocalizations. Sample sizes were n = 8 for Empty + Predator, n = 8 for Empty + Sparrow, n = 9 for Estradiol + Predator, and n = 8 for Estradiol + Sparrow. See Methods for data point losses. **(A)** AMV = medial ventral arcopallium, **(B)** cHP = caudal hippocampus, **(C)** HA = apical hyperpallium, **(D)** NCL = caudolateral nidopallium, **(E)** NCM = caudomedial nidopallium, and **(F)** CMM = caudomedial mesopallium. Error bars represent standard error.

We found a significant overall effect of sound treatment on movement (Figure 2A; $X_{1}^{2}$ = 5.98, *p* = 0.015), where female sparrows exposed to predator calls moved less than females exposed to male sparrow calls (Figure 2B; ANOVA: F_1,30_ = 9.71, *p* = 0.004). Movement was not affected by hormone treatment ( $X_{1}^{2}$ = 0.95, *p* = 0.33) and there was no interaction between hormone and sound treatment ( $X_{1}^{2}$ = 1.56, *p* = 0.21). For feeding duration, there was a significant overall effect of sound treatment (Figure 2C; $X_{1}^{2}$ = 10.36, *p* = 0.0013), and a significant interaction between hormone and sound treatment (Figure 2C; $X_{1}^{2}$ = 8.41, *p* = 0.0037). Females exposed to estradiol implants and sparrow vocalizations spent more time feeding compared to all other treatment groups (Figure 2C; Tukey’s HSD: all *p* < 0.0079). There was also a significant overall effect of hormone treatment on feed duration (Figure 3C; $X_{1}^{2}$ = 4.67, *p* = 0.031); however, this became non-significant after Holm-Bonferroni correction.

### 3.2 ZENK activity

There was a significant effect of hormone treatment on ZENK activity in the HA (Figure 3C; $X_{1}^{2}$ = 5.07, *p* = 0.024), NCL (Figure 3D; $X_{1}^{2}$ = 4.45, *p* = 0.035), and CMM (Figure 3F; $X_{1}^{2}$ = 4.59, *p* = 0.032); however, after Holm-Bonferroni correction for multiple testing all *p*-values became non-significant (all *p* > 0.05). We did not detect an overall effect of sound treatment (all *p* > 0.17), or an interaction between hormone and sound treatment (all *p* > 0.66) in any region of interest, nor did we find an effect of hormone treatment in the AMV, cHP, and NCM (Figures 2A,B,E; all *p* > 0.18). There were no significant correlations between ZENK activity in any of the brain regions of interest and movement or feeding (all Spearman’s coefficients <0.09, all *p* > 0.12).

## 4 Discussion

The overall goal of this research was to understand the effect of hormonal state on behavior and brain activity in response to breeding and predation signals. Female songbirds in breeding condition were previously shown to have higher neuronal activity in response to conspecific song than to neutral tones (Maney et al., 2008; Sanford et al., 2010) and heterospecific song (Lattin et al., 2017b), but there had been no direct comparisons to sounds encoding potential threats. We hypothesized that female songbirds exposed to estradiol may also selectively “tune out” predator calls in favor of conspecific song, but we did not find support for this hypothesis. We instead found support for the null hypothesis, finding that neuronal activity did not differ in females hearing predator calls and male sparrow song, regardless of estradiol exposure. These data suggest female sparrows do not decrease their sensitivity to predator vocalizations while they are in breeding condition, and that this is not an explanation for the previously observed increase in depredation of breeding female songbirds (Slagsvold and Dale, 1996; Götmark et al., 1997; Post and Götmark, 2006). Thus, our data suggest there is no trade-off between females’ auditory sensitivity toward mates and threats, and conspecific and predator cues both elicit a strong increase in neuronal activity in several brain regions.

We predicted that female sparrows exposed to predator calls would show more freezing behavior compared to sparrows exposed to conspecific song. Our findings supported this prediction: female sparrows exposed to predator playback moved less than females exposed to male sparrow song, regardless of hormone treatment. This is likely due to sparrows freezing to reduce attention to themselves while a predator is nearby, as seen in previous avian studies (Borchelt and Ratner, 1973; Quinn and Cresswell, 2005). Freezing behaviors are a common antipredator strategy of many wild animals in response to direct predator signals or the alarm calls of conspecifics (Beani and Dessí-Fulgheri, 1998; Rahlfs and Fichtel, 2010; Bedore et al., 2015). Other antipredator behaviors include vigilance and escape; however, the type of antipredator behavior exhibited may depend on the type of predator signal. For example, red-legged partridges *(Alectoris rufa*) exposed to taxidermy models of terrestrial and aerial predators froze more in response to aerial predators and were most vigilant in response to terrestrial predators (Binazzi et al., 2011). Thus, we may have observed freezing (characterized as less movement in our study) due to the inclusion of aerial predator calls in our recordings.

We gave sparrows estradiol implants to simulate breeding condition. Consistent with previous studies using estradiol implants in female house sparrows (Lattin et al., 2017b), we did not observe copulation solicitation behaviors, one of the main mating behaviors studied in female birds (Searcy and Capp, 1997). However, sparrows that previously had estradiol implants spent more time feeding when exposed to conspecific song. This contradicts many rodent studies where increasing estradiol decreases feeding, and, further, that ovariectomized rodents increase feeding (Butera, 2010). However, estradiol implants increase feeding in Holstein heifers (Lammers et al., 1999), and estradiol-treated female sparrows have high circulating glucose levels, demonstrating that estradiol increases energy mobilization in house sparrows (Lattin et al., 2017b). Altogether, these studies suggest a relationship between estrogen levels and feeding behavior, but the direction of the relationship may vary by species. This relationship may be driven by reproductive strategy and seasonality: both heifers and songbirds are seasonal breeders, whereas laboratory rodents are not. Additionally, we only observed increased feeding behavior in females previously exposed to estradiol hearing conspecific song, not in those hearing predator calls. House sparrows increase their latency to feed in the presence of a predator signal (Seress et al., 2011); therefore, females in breeding condition may suppress feeding behavior in the presence of predator calls to increase vigilance. A trade-off between feeding and vigilance behavior is commonly observed across different species (Dill and Fraser, 1984; Poysa, 1987; Pueta et al., 2016).

Although female sparrows displayed different behavioral responses to predator and conspecific playback, we did not observe differences in ZENK activity in any of our regions of interest. We would typically expect that large differences in behavior would co-occur with differences in neuronal activity in parts of the brain involved in perceiving and responding to threats (e.g., AMV, cHP), which has been seen in other studies (Cross et al., 2013; Zanette et al., 2019; Freeman et al., 2020). Behavioral discrimination of conspecific calls has been positively correlated with activation of specific brain regions in rodents, but not in many other species (Sadananda et al., 2008; Schwarting et al., 2018), and we did not find any significant correlations between ZENK activity in any of our brain regions of interest and movement or feeding behavior. One possible explanation for these results is that regions we did not examine in this study may have been important in responding to predator cues, and these unexamined regions might have differed in ZENK expression. Other possible regions of interest could include the paraventricular nucleus and lateral septum, both of which are involved in regulating emotional and hormonal responses to stressful stimuli (Nagarajan et al., 2014; Smulders, 2021; 2017; Iyer and Tole, 2020). Alternatively, we may have seen differences in IEG activation in response to predator and conspecific playbacks if had we examined expression of a different IEG, for example, c-Fos. Several studies have found differences in the expression of different IEGs in response to the same stimulus (Sewall and Davies, 2017; Kimball et al., 2022). We chose ZENK because it is commonly used to assess neuronal activity in songbirds in response to acoustic stimuli (Maney et al., 2003; Rivera et al., 2019); however, future studies should consider using multiple IEGs to examine the brain’s response to positive, neutral, or aversive auditory cues. Lastly, the inclusion of a neutral frequency-matched tone treatment or silent treatment group may have showed differences in brain responses to conspecific or predator vocalizations relative to neutral tones or silence; however, we were specifically interested in females’ responses to predators relative to conspecifics, so we did not include this type of control.

In conclusion, female sparrows altered their behavior in response to breeding hormones and predator calls, specifically showing increased freezing behavior in response to potentially threatening sounds. Additionally, estradiol-treated female sparrows did not selectively “tune out” predator calls in the same way they can “tune out” neutral tones or heterospecific bird song (Maney et al., 2008; Sanford et al., 2010; Lattin et al., 2017b). These results are compelling because they suggest that different types of sounds may be perceived–and responded to–differently by breeding females, depending on the information content and valence of these sounds. Specifically, sparrows maintain high neuronal responses to signals directly related to reproduction and survival. Together, these behavioral and neural findings indicate that female sparrows are vigilant and responsive to the threat of predation regardless of their breeding condition.

## Data Availability

The original contributions presented in the study are included in the article/supplementary material, further inquiries can be directed to the corresponding author.
